# Crystal structures of three *N*-(pyridine-2-carbon­yl)pyridine-2-carboxamides as potential ligands for supra­molecular chemistry

**DOI:** 10.1107/S2056989021008562

**Published:** 2021-08-24

**Authors:** Xiaowen Xu, Richard Hoogenboom, Kristof Van Hecke

**Affiliations:** a Supramolecular Chemistry Group, Centre of Macromolecular Chemistry (CMaC), Department of Organic and Macromolecular Chemistry, Ghent University, Krijgslaan 281-S4, B-9000 Ghent, Belgium; b XStruct, Department of Chemistry, Ghent University, Krijgslaan 281-S3, B-9000 Ghent, Belgium

**Keywords:** supra­molecular chemistry, *N*-(pyridine-2-carbon­yl)pyridine-2-carboxamide, fluorine moieties, crystal structure

## Abstract

The synthesis and single-crystal X-ray structures of three *N*-(pyridine-2-carbon­yl)pyridine-2-carboxamide imides, with or without F atoms on the 3-position of the pyridine rings are reported, namely, *N*-(pyridine-2-carbon­yl)pyridine-2-carboxamide, C_12_H_9_N_3_O_2_ (**1**), *N*-(3-fluoro­pyridine-2-carbon­yl)pyridine-2-carboxamide, C_12_H_8_FN_3_O_2_ (**2**), and 3-fluoro-*N*-(3-fluoro­pyridine-2-carbon­yl)pyridine-2-carboxamide, C_12_H_7_F_2_N_3_O_2_ (**3**). The above-mentioned compounds were synthesized by a mild, general procedure with an excellent yield, providing straightforward access to symmetrical and/or asymmetrical heterocyclic ureas. The crystal structures of **1** and **2** are isomorphous, showing similar packing arrangements, *i.e.* double layers of parallel (face-to-face) mol­ecules alternating with analogous, but perpendicularly oriented, double layers. In contrast, the crystal structure of **3**, containing a fluoro- group at the 3-position of both pyridine rings, shows mol­ecular arrangements in a longitudinal, tubular manner along the *c* axis, with the aromatic pyridine and carbon­yl/fluorine moieties facing towards each other.

## Abstract 2

The crystal structures of three *N*-(pyridine-2-carbon­yl)pyridine-2-carboxamide ligands, with or without F atoms on the 3-position of the pyridine ring, with potential use in supra­molecular chemistry are reported.

## Chemical context   

*N*-(Pyridine-2-carbon­yl)pyridine-2-carboxamide systems and their derivatives have been shown to be very useful inter­mediates for the construction of mol­ecular building blocks, able to self-assemble into a wide range of super-architectures taking advantage of acceptor–donor–donor–acceptor (ADDA) arrays of hydrogen-bonding sites (Corbin *et al.*, 2001 ▸). Further inter­est in this family of compounds has involved the investigation of their metal coordination complexes, which possess strong luminescence characteristics (Das *et al.*, 2018 ▸), as well as their electrochemical (Gasser *et al.*, 2012 ▸), magnetic (Kajiwara *et al.*, 2010 ▸) and catalytic properties (Chowdhury *et al.*, 2007 ▸). Consequently, the synthesis of *N*-(pyridine-2-carbon­yl)pyridine-2-carboxamide, containing different functional groups, at a large scale and in a high yield is of great importance in the field of supra­molecular chemistry. Previously reported studies have shown the conversion of 2-amino­pyridine to **1** in a single step (Gerchuk & Taits, 1950 ▸; Corbin *et al.*, 2001 ▸). However, the utilized reaction conditions were, to some extent, harsh and the reported yield of the compound was rather low (< 32%), presumably because of the inferior nucleophilicity of the –NH_2_ groups at the 2-position of the pyridine rings. Moreover, the use of this procedure is limited to the synthesis of symmetrical imides. The synthesis of high-yield asymmetrical imides, bearing different functional groups on the pyridine rings, is still challenging.
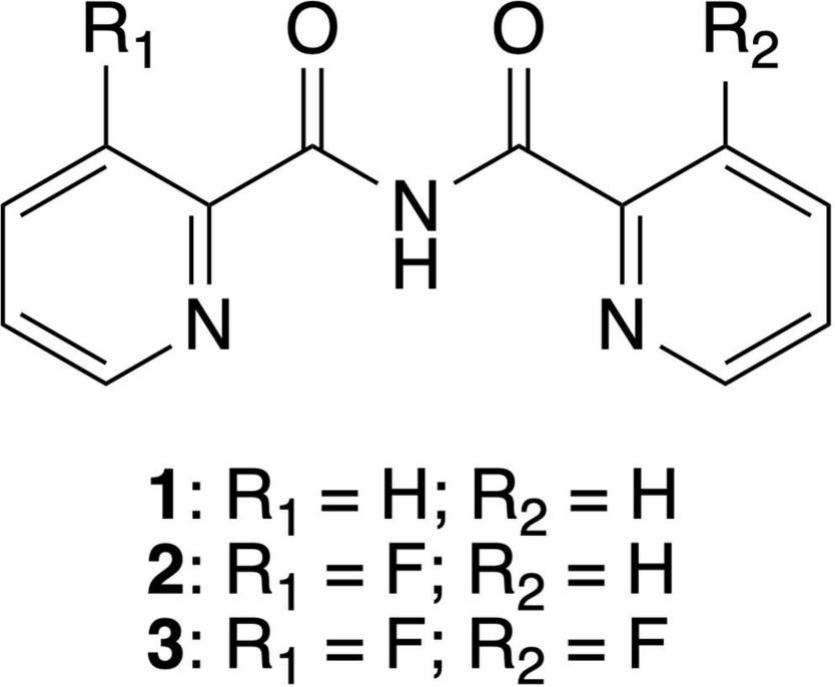


Herein, we report the single-crystal X-ray structural analysis of the imides *N*-(pyridine-2-carbon­yl)pyridine-2-carboxamide (**1**) (*R*_1_ = H, *R*_2_ = H), *N*-(3-fluoro­pyridine-2-carbon­yl)pyridine-2-carboxamide (**2**) (*R*_1_ = F, *R*_2_ = H) and 3-fluoro-*N*-(3-fluoro­pyridine-2-carbon­yl)pyridine-2-carboxamide (**3**) (*R*_1_ = F, *R*_2_ = F), prepared *via* a simple, straightforward synthesis method that does not involve high pressure nor harsh conditions and can be carried out on a large scale.

## Structural commentary   

**Figure 1:**
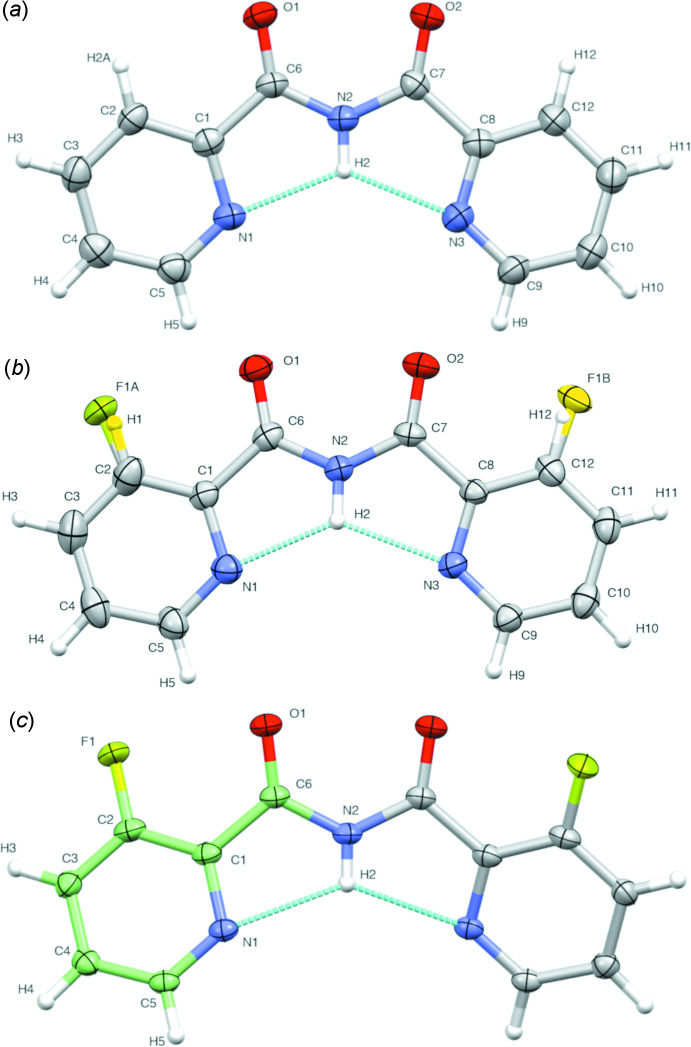
Mol­ecular structures of (*a*) **1**, (*b*) **2** and (*c*) **3**, showing thermal displacement ellipsoids drawn at the 50% probability level and the atom-labelling scheme. The disorder in **2** (*b*) is shown in yellow. The carbon atoms in the asymmetric unit of **3** (*c*) are shown in green. Intra­molecular hydrogen bonds are indicated.

The structure of **1**, although determined at a different temperature of 200 K, has previously been deposited in the CSD (refcode COJNAT; Castaneda & Gabidullin, 2019 ▸). Compound **1** crystallizes in the non-centrosymmetric ortho­rhom­bic space group *Pna*2_1_, with the asymmetric unit consisting of one *N*-(pyridine-2-carbon­yl)pyridine-2-carboxamide mol­ecule. The mol­ecular structure of **1** is found almost completely planar, with a dihedral angle of 6.1 (2)° between the best planes through the two pyridine rings (Fig. 1 ▸ *a*).

**Figure 2:**
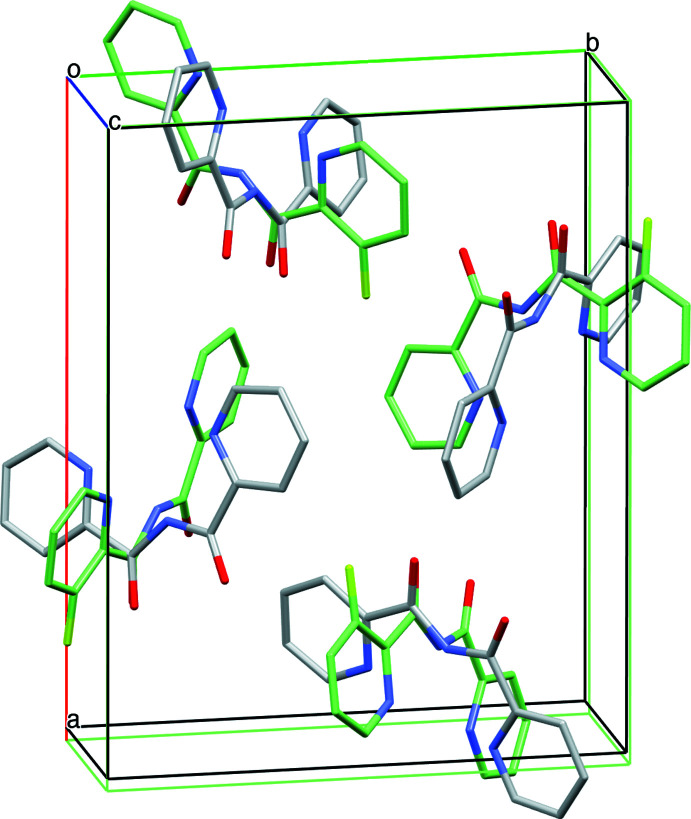
Unit-cell fit of the structures of **1** and **2**, showing a 90° rotation of the mol­ecules of **2** (in green). Hydrogen atoms and disorder of the fluorine atoms are omitted for clarity.

The structure of **2** is isomorphous with **1**, although the 3-fluoro-*N*-(pyridine-2-carbon­yl)pyridine-2-carboxamide mol­ecules are rotated 90° with respect to **1** (Fig. 2 ▸). Similarly to **1**, the asymmetric unit contains one planar 3-fluoro-*N*-(pyridine-2-carbon­yl)pyridine-2-carboxamide mol­ecule, which shows a dihedral angle of 5.2 (2)° between the best planes through the two pyridine rings. Here, the fluoro group is found disordered over both pyridine rings, *i.e.* a transverse disorder by 180° rotation along the axis through the imide N—H function occurs, showing refined occupancy factors of 0.563 (8) and 0.437 (8) for the first (F1*A*) and second fluoro (F1*B*) site, respectively (Fig. 1 ▸ *b*).

Compound **3** crystallizes in the centrosymmetric monoclinic space group *I*2/*a*, with the asymmetric unit consisting of only half of a total 3-fluoro-*N*-(3-fluoro-pyridine-2-carbon­yl)pyridine-2-carboxamide mol­ecule. The second half is generated by symmetry, *i.e.* a twofold axis runs through the N—H imide atoms. In contrast to the previous structures of **1** and **2**, the mol­ecular structure of **3** is not planar, with a dihedral angle of 29.73 (11)° between the best planes through the two pyridine rings (Fig. 1 ▸ *c*).

## Supra­molecular features   

**Figure 3:**
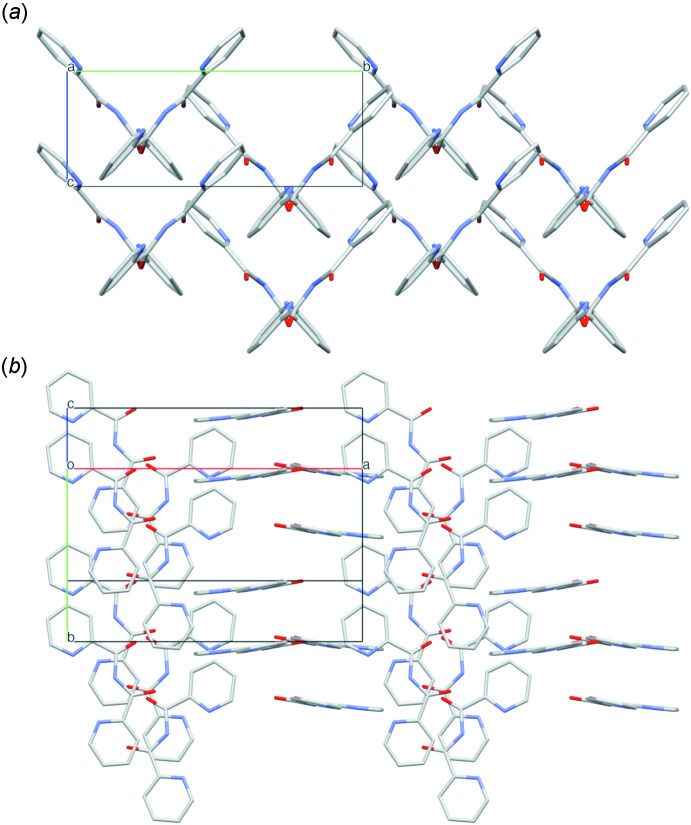
Packing in the structure of **1**, showing (*a*) the perpendicularly oriented mol­ecules, viewed down the *a* axis and (*b*) the double layers of parallel-oriented (face-to-face) mol­ecules, inter­changed with analogous double layers, perpendicular to the former layers.

Despite the presence of two pyridine rings in the mol­ecular structure of **1**, only weak π–π inter­actions are present in the crystal packing, with rather large centroid–centroid distances ranging from 4.969 (2) to 5.497 (2) Å. However, clear C=O⋯π contacts are observed in the crystal packing [C6—O1⋯*Cg*1(*x*, *y*, −1 + *z*) = 3.861 (3) Å; *Cg*1 is the centroid of the C1–C5/N1 ring]. Intra­molecular potential hydrogen bonds are found between the imide N2—H2 hydrogen atom and both pyridine nitro­gen atoms [N2—H2⋯N1 = 2.15 (6) Å; N2—H2⋯N3 = 2.15 (5) Å], while non-classical inter­molecular hydrogen bonds can be observed between the first pyridine rings and carbonyl O2 atoms of symmetry-equivalent mol­ecules [C3—H3⋯O2^i^ = 2.48 Å; symmetry code: (i) 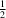 − *x*, 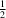 + *y*, −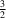 + *z*], while these first pyridine rings are further connected to each other *via* similar hydrogen bonds with the pyridine N1 atoms [C5—H5⋯N1^ii^ = 2.51 Å; symmetry code: (ii) −*x*, 1 − *y*, −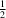 + *z*] (Table 1 ▸). As such, in the packing, double layers of parallel (face-to-face) mol­ecules of **1** are observed, parallel with the (100) plane, alternating with analogous double layers, oriented perpendicular to the former layers (Fig. 3 ▸).

**Figure 4:**
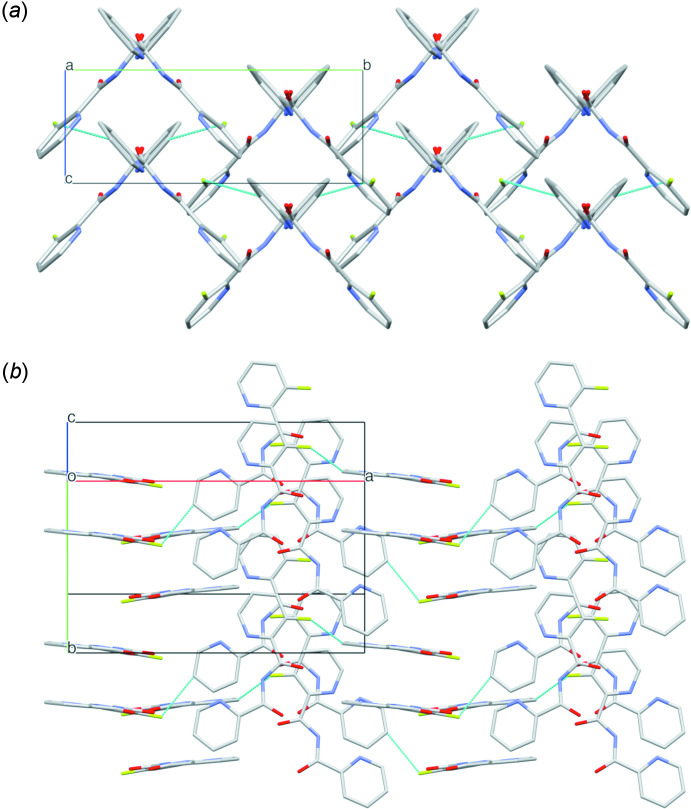
Packing in the structure of **2**, showing (*a*) the perpendicularly oriented mol­ecules, viewed down the *a* axis and (*b*) the double layers of parallel-oriented (face-to-face) mol­ecules, inter­changed with analogous double layers, perpendicular to the former layers. C10—H10⋯F1*A* hydrogen bonds are indicated. Hydrogen atoms and disorder of the fluorine atoms are omitted for clarity.

For the structure of **2**, analogous to **1**, only weak π–π inter­actions are present in the crystal packing between the 3-fluoro-pyridine rings, with centroid–centroid distances in the range 4.915 (3) to 5.473 (3) Å, while C=O⋯π contacts are also observed in the crystal packing [C6—O1⋯*Cg*1(*x*, *y*, −1 + *z)*= 3.865 (4) Å; *Cg*1 is the centroid of the C1–C5/N1 ring]. Analogous to **1**, intra­molecular potential hydrogen bonds between the imide N2—H2 hydrogen atom and both pyridine nitro­gen atoms are observed [N2—H2⋯N1 = 2.16 (6) Å; N2—H2⋯N3 = 2.11 (6) Å], while non-classical inter­molecular hydrogen bonds occur between the first pyridine rings and carbonyl O2 atoms of symmetry-equivalent mol­ecules [C3—H3⋯O2^i^ = 2.43 Å; symmetry code: (i) 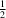 − *x*, 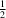 + *y*, 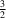 + *z*], while these first pyridine rings are further connected to each other *via* similar hydrogen bonds with the pyridine N1 atoms [C5—H5⋯N1^ii^ = 2.53 Å; symmetry code: (ii) 1 − *x*, 2 − *y*, 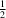 + *z*]. Additionally, C—H⋯F hydrogen bonds are observed with the two disordered fluorine moieties [C3—H3⋯F1*B* ^i^ = 2.40 Å; C10—H10⋯F1*A* ^iii^ = 2.45 Å; symmetry code: (iii) 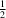 + *x*, 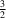 − *y*, −1 + *z*] (Table 2 ▸). However, in the packing, analogous to **1**, alternating double layers of parallel (face-to-face) mol­ecules of **2** are observed, parallel with the (100) plane (Fig. 4 ▸). Hence, the extra C—H⋯F bonds do not alter the overall architecture.

**Figure 5:**
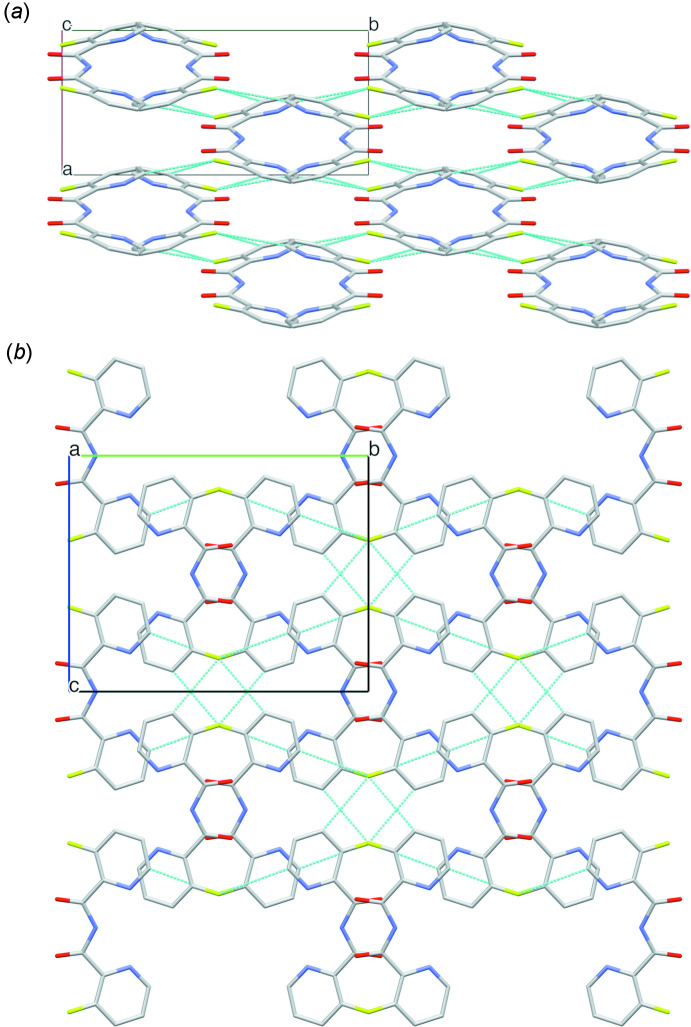
Packing in the structure of **3**, showing (*a*) the longitudinal tubular arrangement of the mol­ecules along the *c* axis and (*b*) the aromatic pyridine and the carbon­yl/fluorine moieties facing towards each other. C5—H5⋯F1 and C3—H3⋯F1 hydrogen bonds are indicated. Hydrogen atoms are omitted for clarity.

For **3**, besides weak π–π inter­actions between the pyridine rings [centroid–centroid distances in the range 4.3776 (13)–5.9437 (13) Å], one strong π–π contact is observed between the pyridine ring and its symmetry-equivalent [*Cg*⋯*Cg*(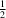 − *x*, 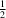 − *y*, 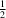 − *z*) = 3.6334 (13) Å; *Cg* is the centroid of the C1–C5/N1 ring]. Analogous to **1** and **2**, intra­molecular potential hydrogen bonds are observed between the imide N2—H2 hydrogen atom and the pyridine nitro­gen atom [N2—H2⋯N1 = 2.265 (15) Å], while non-classical inter­molecular hydrogen bonds between the pyridine rings and carbonyl O1 atoms of symmetry-equivalent mol­ecules are found [C4—H4⋯O1^ii^ = 2.49 Å; symmetry code: (ii) −*x*, −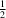 + *y*, 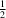 − *z*] (Table 3 ▸). Additionally, although significantly longer, other hydrogen bonds are formed between the pyridine ring and the carbonyl O1 atom [C5—H5⋯O1^ii^ = 2.61 Å] and C—H⋯F hydrogen bonds are observed with the fluorine moieties [C5—H5⋯F1^ii^ = 2.66 Å; C3—H3⋯F1^iii^ = 2.58 Å; symmetry codes: (iii) −*x*, 1 − *y*, −*z*]. This gives rise to a different packing assembly, *i.e.* the mol­ecules are arranged in a longitudinal, tubular manner along the *c*-axis direction, while the aromatic pyridine and the carbon­yl/fluorine moieties, face towards each other (Fig. 5 ▸).

## Database survey   

A survey of compounds related to **1**, **2** and **3**, deposited with the Cambridge Structural Database (CSD 2021.1, version 5.42 updates May 2021; Groom *et al.*, 2016 ▸) resulted in three other compounds with refcodes COJNAT, WUXQOW and ZAVVAV.

As previously mentioned, COJNAT (Castaneda & Gabidullin, 2019 ▸) represents the same structure as **1**, although determined at 200 K. When fitting the mol­ecular structures of COJNAT and **1**, an r.m.s.d. of 0.0107 Å is obtained.

The structure with refcode WUXQOW (Sahu *et al.*, 2010 ▸) represents an analogous structure to **1**, but featuring quinoline moieties instead of pyridine rings, *i.e. N,N*-bis­(quinolin-2-ylcarbon­yl)amine. Similarly to **1**, the mol­ecular structure is also found to be almost completely planar, with a dihedral angle of 1.34 (4)° between the best planes through the two quinoline moieties.

The structure with refcode ZAVVAV (Zebret *et al.*, 2012 ▸) represents another *N*-(pyridine-2-carbon­yl)pyridine-2-carboxamide system, in this case featuring two meth­oxy substituents, one on each pyridine ring, *i.e.* methyl 6-({[6-(meth­oxy­carbon­yl)pyridin-2-yl]carbon­yl}carbamo­yl)pyridine-2-carboxyl­ate. Here, because of steric hindrance of the substituents, the planes defined by the two pyridine rings are distorted by 14.52 (11)°.

## Synthesis and crystallization   

The known compound **1** was prepared in excellent yield by the reaction between 2-pyridine­carbonyl chloride and 2-pyri­dine­carboxamide under mild conditions. By introducing a fluoro group at the 3-position of 2-pyridine­carbonyl chloride and/or 2-pyridine­carboxamide, the new compounds **2** and **3** could be obtained, also in excellent yield. Details for the synthesis of the precursors and the products are given below. Unless otherwise stated, all reagents were used as received.

**3-Fluoro­pyridine-2-carb­oxy­lic acid**

The preparation of 3-fluoro­pyridine-2-carb­oxy­lic acid was performed according to a previously reported procedure (Eller *et al.*, 2006 ▸). Commercially available lithium 3-fluoro­picolinate (1.47 g, 10 mmol) was recrystallized from a mixture of EtOH–H_2_O (9:1), which was acidified with several drops of concentrated HCl (36.5%) to afford 3-fluoro­pyridine-2-carb­oxy­lic acid. Yield: 91%. ^1^H NMR (300 MHz, DMSO-*d*_6_) δ 8.49 (*d*, *J* = 4.4 Hz, 1H), 7.94–7.81 (*m*, 1H), 7.64–7.70 (*m*, 1H). ^13^C NMR (101 MHz, DMSO-*d*_6_) δ 164.35, 159.27, 145.26, 138.65, 128.27, 125.59.

**2-Pyridine­carbonyl chloride**

The preparation of 2-pyridine­carbonyl chloride was performed according to a previously reported procedure (Aluri *et al.*, 2011 ▸). 2-Pyridine­carb­oxy­lic acid (1.23 g, 10 mmol) and SOCl_2_ (11.9 g, 100 mmol) were dissolved in 100 ml of dry toluene with 10 drops of DMF. The reaction mixture was refluxed at 383.15 K for 3 h. The reaction mixture was cooled to room temperature and the solvent was removed under reduced pressure. The resulting viscous residue was used directly in the next step without further purification.

**3-Fluoro­pyridine-2-carbonyl chloride**

The preparation of 3-fluoro­pyridine-2-carbonyl chloride was performed according to a previously reported procedure (Aluri *et al.*, 2011 ▸). 3-Fluoro­pyridin-2-carb­oxy­lic acid (1.41 g, 10 mmol) and SOCl_2_ (11.9 g, 100 mmol) were dissolved in 100 ml of dry toluene with 10 drops of DMF. The reaction mixture was refluxed at 383 K for 3 h. The reaction mixture was cooled to room temperature and the solvent was removed under reduced pressure. The resulting viscous residue was used directly in the next step without further purification.

**2-Pyridine­carboxamide**

The preparation of 2-pyridine­carboxamide was performed according to a previously reported procedure (Cai *et al.*, 2014 ▸). 20 ml of NH_3_/methanol solution (NH_3_ *ca* 7 *N* in methanol solution) was slowly added to 2-pyridine­carbonyl chloride at 273 K under stirring. The resulting reaction mixture was allowed to warm to room temperature and stirred overnight. The solvent was removed under reduced pressure and the residue was purified by a silica column with an eluent of hexa­ne/ethyl acetate (5/1) to afford the product. Yield: 88%. ^1^H NMR (300 MHz, DMSO-*d*_6_) δ 8.63 (*d*, *J* = 4.7 Hz, 1H), 8.11 (*s*, 1H), 8.06–7.94 (*m*, 2H), 7.64 (*s*, 1H), 7.63–7.55 (*m*, 1H).

**3-Fluoro­pyridin-2-carboxamide**

20 ml of NH_3_/methanol (NH_3_ *ca* 7 *N* in methanol solution) was added slowly to 3-fluoro­pyridin-2-carbonyl chloride at 273 K under stirring. The resulting reaction mixture was allowed to warm to room temperature and stirred overnight. The solvent was removed under reduced pressure and the residue was purified by silica column with an eluent of hexa­ne/ethyl acetate (5/1) to afford the product. Yield 85%. ^1^H NMR (300 MHz, CDCl_3_) δ 8.34 (*dt*, *J* = 4.2, 1.4 Hz, 1H), 7.63 (*s*, 1H), 7.54–7.40 (*m*, 2H), 6.30 (*s*, 1H). ^13^C NMR (101 MHz, CDCl_3_) δ 164.96, 164.91, 158.20, 144.12, 144.07, 137.26, 128.42, 128.37, 126.36, 126.16.

***N*** **-(Pyridine-2-carbon­yl)pyridine-2-carboxamide (1)**

2-Pyridine­carbonyl chloride (212.32 mg, 1.5 mmol) and 2-pyridine­carboxamide (170.98 mg, 1.4 mmol) were dissolved in toluene (20 ml). The resulting reaction mixture was refluxed at 383 K overnight. The solvent was removed under reduced pressure and the residue was purified by a silica column with an eluent of hexa­ne/ethyl acetate (3/1) to afford the product. Yield: 91%. ^1^H NMR (300 MHz, CDCl_3_) δ 13.03 (*s*, 1H), 8.75 (*ddd*, *J* = 4.8, 1.7, 0.9 Hz, 2H), 8.35 (*dt*, *J* = 7.9, 1.1 Hz, 2H), 7.94 (*td*, *J* = 7.7, 1.7 Hz, 2H), 7.56 (*ddd*, *J* = 7.6, 4.8, 1.2 Hz, 2H). ^13^C NMR (101 MHz, CDCl_3_) δ 162.65, 149.15, 148.67, 137.73, 127.50, 123.49.

***N*** **-(3-Fluoro­pyridine-2-carbon­yl)pyridine-2-carboxamide (2)**

3-Fluoro­pyridin-2-carboxamide (238.47 mg, 1.5 mmol) and 2-pyridine­carboxamide (170.98 mg, 1.4 mmol) were dissolved in toluene (20 ml). The resulting reaction mixture was refluxed at 383 K overnight. The solvent was removed under reduced pressure and the residue was purified by a silica column with an eluent of hexa­ne/ethyl acetate (3/1) to afford the product. Yield: 89%. ^1^H NMR (300 MHz, DMSO-*d*_6_) δ 12.72 (*s*, 1H), 8.81 (*ddd*, *J* = 4.8, 1.6, 0.9 Hz, 1H), 8.66 (*dt*, *J* = 4.5, 1.4 Hz, 1H), 8.22 (*dt*, *J* = 7.8, 1.1 Hz, 1H), 8.13 (*td*, *J* = 7.7, 1.7 Hz, 1H), 8.02 (*ddd*, *J* = 11.3, 8.5, 1.2 Hz, 1H), 7.92–7.85 (*m*, 1H), 7.78 (*ddd*, *J* = 7.5, 4.8, 1.3 Hz, 1H). ^13^C NMR (101 MHz, DMSO-*d*_6_) 161.88, 160.91, 159.53, 159.47, 158.21, 148.97, 148.16, 144.99, 144.93, 138.66, 135.97, 135.92, 130.72, 130.67, 128.35, 127.45, 127.26, 122.94.

**3-Fluoro-** ***N*** **-(3-fluoro­pyridine-2-carbon­yl)pyridine-2-carb­ox­amide (3)**

3-Fluoro­pyridin-2-carboxamide (238.47 mg, 1.5 mmol) and 3-fluoro­pyridin-2-carbonyl chloride (196.04 mg, 1.4 mmol) were dissolved in toluene (20 ml). The resulting reaction mixture was refluxed at 383 K overnight. The solvent was removed under reduced pressure and the residue was purified by a silica column with an eluent of hexa­ne/ethyl acetate (3/1) to afford the product. Yield: 80%. ^1^H NMR (300 MHz, DMSO-*d*_6_) δ 12.53 (*s*, 1H), 8.64 (*dt*, *J* = 4.5, 1.4 Hz, 2H), 8.02 (*ddd*, *J* = 11.3, 8.5, 1.2 Hz, 2H), 7.91–7.80 (*m*, 2H). ^13^C NMR (101 MHz, DMSO-*d*_6_) 160.75, 159.72, 159.66, 158.05, 156.16, 144.97, 144.92, 136.12, 136.08, 130.62, 130.56, 127.36, 127.17.

Crystals of **1**, **2**, and **3**, suitable for single-crystal X-ray diffraction analysis were prepared by slow evaporation of a 10 mg ml^−1^ aceto­nitrile solution at room temperature. All crystals appeared as colourless blocks.

## Refinement   

Crystal data, data collection and structure refinement details are summarized in Table 4 ▸. For all structures, the imide N—H hydrogen atoms could be located from a difference electron-density Fourier map, and were further refined with isotropic temperature factors fixed at 1.2 times *U*_eq_ of the parent atoms.

For the structure of **2**, the 3-fluoro­pyridine atom is disordered at both pyridine sites, showing final occupancy factors of 0.563 (8) and 0.437 (8), for the first and second site, respectively.

## Supplementary Material

Crystal structure: contains datablock(s) global, 1, 2, 3. DOI: 10.1107/S2056989021008562/vm2252sup1.cif

Supporting information file. DOI: 10.1107/S2056989021008562/vm22521sup2.cml

Supporting information file. DOI: 10.1107/S2056989021008562/vm22522sup3.cml

Supporting information file. DOI: 10.1107/S2056989021008562/vm22523sup4.cml

CCDC references: 2103652, 2103651, 2103650

Additional supporting information:  crystallographic information; 3D view; checkCIF report

## Figures and Tables

**Table 1:** Hydrogen-bond geometry (Å, °) for **1** [Chem scheme1]

*D*—H⋯*A*	*D*—H	H⋯*A*	*D*⋯*A*	*D*—H⋯*A*
N2—H2⋯N1	0.90 (5)	2.15 (6)	2.614 (5)	111 (4)
N2—H2⋯N3	0.90 (5)	2.15 (5)	2.637 (4)	113 (5)
C3—H3⋯O2^i^	0.95	2.48	3.343 (5)	152
C5—H5⋯N1^ii^	0.95	2.51	3.393 (5)	154

Symmetry codes: (i) -x+{\script{1\over 2}}, y+{\script{1\over 2}}, z+{\script{3\over 2}}; (ii) -x, -y+1, z-{\script{1\over 2}}.

**Table 2:** Hydrogen-bond geometry (Å, °) for **2** [Chem scheme1]

*D*—H⋯*A*	*D*—H	H⋯*A*	*D*⋯*A*	*D*—H⋯*A*
N2—H2⋯N1	0.92 (5)	2.16 (6)	2.614 (6)	109 (4)
N2—H2⋯N3	0.92 (5)	2.11 (6)	2.622 (5)	114 (5)
C3—H3⋯O2^i^	0.95	2.43	3.320 (6)	156
C3—H3⋯F1*B* ^i^	0.95	2.40	3.049 (8)	125
C5—H5⋯N1^ii^	0.95	2.53	3.420 (6)	156
C10—H10⋯F1*A* ^iii^	0.95	2.45	3.169 (7)	132

Symmetry codes: (i) -x+{\script{1\over 2}}, y+{\script{1\over 2}}, z+{\script{3\over 2}}; (ii) -x+1, -y+2, z+{\script{1\over 2}}; (iii) x+{\script{1\over 2}}, -y+{\script{3\over 2}}, z-1.

**Table 3:** Hydrogen-bond geometry (Å, °) for **3** [Chem scheme1]

*D*—H⋯*A*	*D*—H	H⋯*A*	*D*⋯*A*	*D*—H⋯*A*
N2—H2⋯N1	0.84 (4)	2.27 (2)	2.671 (2)	110 (1)
N2—H2⋯N1^i^	0.84 (4)	2.27 (2)	2.671 (2)	110 (1)
C4—H4⋯O1^ii^	0.95	2.49	3.135 (3)	125
C5—H5⋯O1^ii^	0.95	2.61	3.207 (3)	122
C3—H3⋯F1^iii^	0.95	2.58	3.398 (3)	145
C5—H5⋯F1^ii^	0.95	2.66	3.604 (3)	176

Symmetry codes: (i) -x+{\script{1\over 2}}, y, -z+1; (ii) -x, y-{\script{1\over 2}}, -z+{\script{1\over 2}}; (iii) -x, -y+1, -z.

**Table 4:** Experimental details

	**1**	**2**	**3**
Crystal data			
Chemical formula	C_12_H_9_N_3_O_2_	C_12_H_8_FN_3_O_2_	C_12_H_7_F_2_N_3_O_2_
*M*_r_	227.22	245.21	263.21
Crystal system, space group	Orthorhombic, *P* *n* *a*2_1_	Orthorhombic, *P* *n* *a*2_1_	Monoclinic, *I*2/*a*
Temperature (K)	100	100	100
*a*, *b*, *c* (Å)	16.2689 (6), 12.8086 (7), 4.9983 (2)	16.6058 (10), 12.9096 (7), 4.9153 (3)	6.7062 (3), 14.1190 (5), 11.2074 (5)
α, β, γ (°)	90, 90, 90	90, 90, 90	90, 97.140 (4), 90
*V* (Å^3^)	1041.56 (8)	1053.71 (11)	1052.94 (8)
*Z*	4	4	4
Radiation type	Cu *K*α	Cu *K*α	Cu *K*α
μ (mm^−1^)	0.85	1.03	1.22
Crystal size (mm)	0.20 × 0.12 × 0.06	0.26 × 0.10 × 0.05	0.11 × 0.09 × 0.06

Data collection			
Diffractometer	SuperNova, Dual, Cu at zero, Atlas	SuperNova, Dual, Cu at zero, Atlas	SuperNova, Dual, Cu at zero, Atlas
Absorption correction	Gaussian (*CrysAlis PRO*; Rigaku OD, 2015 ▸)	Gaussian (*CrysAlis PRO*; Rigaku OD, 2015 ▸)	Gaussian (*CrysAlis PRO*; Rigaku OD, 2015 ▸)
*T*_min_, *T*_max_	0.187, 0.563	0.983, 0.995	0.993, 0.996
No. of measured, independent and observed [*I* > 2σ(*I*)] reflections	8626, 2028, 1831	5774, 1798, 1567	5200, 1083, 856
*R*_int_	0.076	0.054	0.069
(sin θ/λ)_max_ (Å^−1^)	0.627	0.629	0.628

Refinement			
*R*[*F*^2^ > 2σ(*F* ^2^)], *wR*(*F* ^2^), *S*	0.060, 0.170, 1.07	0.055, 0.152, 1.03	0.055, 0.161, 1.04
No. of reflections	2028	1798	1083
No. of parameters	157	176	88
No. of restraints	1	1	0
H-atom treatment	H atoms treated by a mixture of independent and constrained refinement	H atoms treated by a mixture of independent and constrained refinement	H atoms treated by a mixture of independent and constrained refinement
Δρ_max_, Δρ_min_ (e Å^−3^)	0.32, −0.30	0.28, −0.28	0.29, −0.32
Absolute structure	Flack *x* determined using 673 quotients [(*I* ^+^)−(*I* ^−^)]/[(*I* ^+^)+(*I* ^−^)] (Parsons *et al.*, 2013 ▸)	Flack *x* determined using 450 quotients [(*I* ^+^)−(*I* ^−^)]/[(*I* ^+^)+(*I* ^−^)] (Parsons *et al.*, 2013 ▸)	–
Absolute structure parameter	0.0 (3)	0.2 (3)	–

Computer programs: *CrysAlis PRO* (Rigaku OD, 2015 ▸), *SHELXS* (Sheldrick, 2008 ▸), *SHELXL* (Sheldrick, 2015 ▸) and *OLEX2* (Dolomanov *et al.*, 2009 ▸).

## supplementary crystallographic information

### N-(Pyridine-2-carbonyl)pyridine-2-carboxamide (1) Crystal data

**Table:**

C_12_H_9_N_3_O_2_	*D*_x_ = 1.449 Mg m^−^^3^
*M**_r_* = 227.22	Cu *K*α radiation, λ = 1.54184 Å
Orthorhombic, *P**n**a*2_1_	Cell parameters from 4719 reflections
*a* = 16.2689 (6) Å	θ = 4.2–74.0°
*b* = 12.8086 (7) Å	µ = 0.85 mm^−^^1^
*c* = 4.9983 (2) Å	*T* = 100 K
*V* = 1041.56 (8) Å^3^	Block, clear colourless
*Z* = 4	0.20 × 0.12 × 0.06 mm
*F*(000) = 472	

### N-(Pyridine-2-carbonyl)pyridine-2-carboxamide (1) Data collection

**Table:**

SuperNova, Dual, Cu at zero, Atlas diffractometer	2028 independent reflections
Radiation source: micro-focus sealed X-ray tube, SuperNova (Cu) X-ray Source	1831 reflections with *I* > 2σ(*I*)
Mirror monochromator	*R*_int_ = 0.076
Detector resolution: 10.4839 pixels mm^-1^	θ_max_ = 75.3°, θ_min_ = 4.4°
ω scans	*h* = −14→20
Absorption correction: gaussian (CrysAlisPro; Rigaku OD, 2015)	*k* = −15→15
*T*_min_ = 0.187, *T*_max_ = 0.563	*l* = −5→6
8626 measured reflections	

### N-(Pyridine-2-carbonyl)pyridine-2-carboxamide (1) Refinement

**Table:**

Refinement on *F*^2^	Hydrogen site location: mixed
Least-squares matrix: full	H atoms treated by a mixture of independent and constrained refinement
*R*[*F*^2^ > 2σ(*F*^2^)] = 0.060	*w* = 1/[σ^2^(*F*_o_^2^) + (0.1236*P*)^2^ + 0.0215*P*] where *P* = (*F*_o_^2^ + 2*F*_c_^2^)/3
*wR*(*F*^2^) = 0.170	(Δ/σ)_max_ < 0.001
*S* = 1.07	Δρ_max_ = 0.32 e Å^−^^3^
2028 reflections	Δρ_min_ = −0.30 e Å^−^^3^
157 parameters	Absolute structure: Flack *x* determined using 673 quotients [(*I*^+^)-(*I*^-^)]/[(*I*^+^)+(*I*^-^)] (Parsons *et al.*, 2013)
1 restraint	Absolute structure parameter: 0.0 (3)
Primary atom site location: structure-invariant direct methods	

### N-(Pyridine-2-carbonyl)pyridine-2-carboxamide (1) Special details

**Table:**

Geometry. All esds (except the esd in the dihedral angle between two l.s. planes) are estimated using the full covariance matrix. The cell esds are taken into account individually in the estimation of esds in distances, angles and torsion angles; correlations between esds in cell parameters are only used when they are defined by crystal symmetry. An approximate (isotropic) treatment of cell esds is used for estimating esds involving l.s. planes.

### N-(Pyridine-2-carbonyl)pyridine-2-carboxamide (1) Fractional atomic coordinates and isotropic or equivalent isotropic displacement parameters (Å^2^)

**Table:**

	*x*	*y*	*z*	*U*_iso_*/*U*_eq_	
O1	0.29400 (14)	0.3937 (2)	0.2999 (6)	0.0362 (7)	
O2	0.22855 (16)	0.2512 (2)	0.7000 (6)	0.0343 (7)	
N1	0.10762 (18)	0.4562 (3)	−0.0277 (7)	0.0301 (7)	
N2	0.16131 (17)	0.3339 (2)	0.3528 (7)	0.0299 (7)	
N3	0.01890 (19)	0.2587 (3)	0.5153 (7)	0.0335 (8)	
C1	0.1883 (2)	0.4642 (3)	0.0217 (8)	0.0288 (8)	
C2	0.2393 (2)	0.5328 (3)	−0.1112 (8)	0.0322 (8)	
H2A	0.296091	0.536690	−0.068626	0.039*	
C3	0.2060 (2)	0.5956 (3)	−0.3071 (9)	0.0372 (9)	
H3	0.239464	0.643907	−0.401515	0.045*	
C4	0.1232 (2)	0.5872 (3)	−0.3640 (9)	0.0372 (9)	
H4	0.098806	0.628601	−0.500275	0.045*	
C5	0.0767 (2)	0.5170 (3)	−0.2176 (8)	0.0344 (8)	
H5	0.019566	0.512061	−0.255151	0.041*	
C6	0.2213 (2)	0.3943 (3)	0.2372 (8)	0.0279 (8)	
C7	0.1662 (2)	0.2696 (3)	0.5753 (8)	0.0284 (8)	
C8	0.0840 (2)	0.2245 (3)	0.6517 (8)	0.0290 (8)	
C9	−0.0551 (2)	0.2220 (3)	0.5853 (10)	0.0361 (9)	
H9	−0.101987	0.245492	0.489110	0.043*	
C10	−0.0664 (2)	0.1513 (3)	0.7923 (9)	0.0371 (9)	
H10	−0.119977	0.127904	0.838484	0.045*	
C11	0.0010 (3)	0.1157 (4)	0.9290 (9)	0.0410 (10)	
H11	−0.004963	0.066767	1.070577	0.049*	
C12	0.0785 (2)	0.1526 (3)	0.8565 (9)	0.0368 (9)	
H12	0.126414	0.128826	0.946242	0.044*	
H2	0.110 (3)	0.341 (4)	0.286 (12)	0.044*	

### N-(Pyridine-2-carbonyl)pyridine-2-carboxamide (1) Atomic displacement parameters (Å^2^)

**Table:**

	*U*^11^	*U*^22^	*U*^33^	*U*^12^	*U*^13^	*U*^23^
O1	0.0224 (11)	0.0457 (15)	0.0404 (17)	0.0004 (11)	−0.0002 (11)	0.0017 (13)
O2	0.0291 (12)	0.0387 (14)	0.0352 (15)	0.0032 (10)	−0.0065 (10)	0.0041 (12)
N1	0.0235 (13)	0.0340 (15)	0.0328 (17)	0.0006 (11)	−0.0019 (12)	−0.0011 (13)
N2	0.0235 (13)	0.0350 (15)	0.0312 (17)	0.0004 (11)	−0.0032 (12)	0.0022 (13)
N3	0.0286 (14)	0.0353 (16)	0.0365 (19)	−0.0011 (11)	0.0011 (13)	0.0023 (15)
C1	0.0270 (15)	0.0290 (16)	0.0302 (19)	0.0025 (13)	0.0024 (14)	−0.0040 (14)
C2	0.0270 (16)	0.0333 (18)	0.036 (2)	−0.0007 (13)	0.0064 (15)	−0.0024 (16)
C3	0.0379 (19)	0.0334 (18)	0.040 (2)	0.0007 (15)	0.0100 (17)	0.0029 (17)
C4	0.0424 (19)	0.0361 (18)	0.033 (2)	0.0081 (17)	0.0016 (17)	−0.0015 (16)
C5	0.0308 (16)	0.0393 (18)	0.033 (2)	0.0036 (15)	0.0007 (16)	−0.0005 (17)
C6	0.0214 (15)	0.0318 (17)	0.0304 (19)	0.0016 (12)	−0.0011 (13)	−0.0024 (15)
C7	0.0303 (16)	0.0281 (16)	0.0269 (18)	0.0034 (13)	−0.0011 (14)	0.0002 (14)
C8	0.0283 (16)	0.0296 (17)	0.0292 (19)	0.0010 (13)	−0.0011 (13)	−0.0032 (15)
C9	0.0262 (16)	0.0390 (19)	0.043 (2)	−0.0019 (15)	0.0023 (16)	0.0022 (16)
C10	0.0350 (17)	0.0367 (18)	0.040 (2)	−0.0063 (15)	0.0072 (16)	−0.0006 (17)
C11	0.043 (2)	0.041 (2)	0.038 (3)	−0.0062 (16)	0.0010 (18)	0.0060 (18)
C12	0.0346 (17)	0.041 (2)	0.035 (2)	−0.0004 (15)	−0.0030 (15)	0.0069 (17)

### N-(Pyridine-2-carbonyl)pyridine-2-carboxamide (1) Geometric parameters (Å, º)

**Table:**

O1—C6	1.224 (4)	C3—C4	1.380 (6)
O2—C7	1.214 (4)	C4—H4	0.9500
N1—C1	1.339 (4)	C4—C5	1.385 (6)
N1—C5	1.327 (5)	C5—H5	0.9500
N2—C6	1.373 (5)	C7—C8	1.506 (5)
N2—C7	1.385 (5)	C8—C12	1.380 (6)
N2—H2	0.90 (5)	C9—H9	0.9500
N3—C8	1.333 (5)	C9—C10	1.387 (6)
N3—C9	1.339 (5)	C10—H10	0.9500
C1—C2	1.379 (5)	C10—C11	1.370 (6)
C1—C6	1.499 (5)	C11—H11	0.9500
C2—H2A	0.9500	C11—C12	1.394 (6)
C2—C3	1.378 (6)	C12—H12	0.9500
C3—H3	0.9500		
			
C5—N1—C1	117.3 (3)	O1—C6—C1	122.3 (3)
C6—N2—C7	129.2 (3)	N2—C6—C1	112.6 (3)
C6—N2—H2	116 (3)	O2—C7—N2	125.1 (3)
C7—N2—H2	114 (3)	O2—C7—C8	122.5 (3)
C8—N3—C9	117.8 (4)	N2—C7—C8	112.4 (3)
N1—C1—C2	123.3 (4)	N3—C8—C7	116.7 (3)
N1—C1—C6	115.9 (3)	N3—C8—C12	123.1 (4)
C2—C1—C6	120.7 (3)	C12—C8—C7	120.1 (3)
C1—C2—H2A	120.7	N3—C9—H9	118.6
C3—C2—C1	118.6 (3)	N3—C9—C10	122.9 (4)
C3—C2—H2A	120.7	C10—C9—H9	118.6
C2—C3—H3	120.5	C9—C10—H10	120.5
C2—C3—C4	119.0 (4)	C11—C10—C9	118.9 (4)
C4—C3—H3	120.5	C11—C10—H10	120.5
C3—C4—H4	120.8	C10—C11—H11	120.6
C3—C4—C5	118.3 (4)	C10—C11—C12	118.7 (4)
C5—C4—H4	120.8	C12—C11—H11	120.6
N1—C5—C4	123.5 (4)	C8—C12—C11	118.6 (4)
N1—C5—H5	118.3	C8—C12—H12	120.7
C4—C5—H5	118.3	C11—C12—H12	120.7
O1—C6—N2	125.1 (3)		
			
O2—C7—C8—N3	173.6 (4)	C3—C4—C5—N1	−1.0 (6)
O2—C7—C8—C12	−5.5 (5)	C5—N1—C1—C2	1.0 (6)
N1—C1—C2—C3	−0.8 (6)	C5—N1—C1—C6	−179.9 (3)
N1—C1—C6—O1	179.4 (4)	C6—N2—C7—O2	−4.7 (6)
N1—C1—C6—N2	−1.1 (5)	C6—N2—C7—C8	174.7 (3)
N2—C7—C8—N3	−5.8 (5)	C6—C1—C2—C3	−179.8 (3)
N2—C7—C8—C12	175.2 (4)	C7—N2—C6—O1	7.6 (6)
N3—C8—C12—C11	−1.6 (6)	C7—N2—C6—C1	−171.9 (3)
N3—C9—C10—C11	−1.1 (7)	C7—C8—C12—C11	177.4 (4)
C1—N1—C5—C4	−0.1 (6)	C8—N3—C9—C10	0.3 (6)
C1—C2—C3—C4	−0.3 (6)	C9—N3—C8—C7	−178.0 (3)
C2—C1—C6—O1	−1.5 (5)	C9—N3—C8—C12	1.0 (6)
C2—C1—C6—N2	178.0 (3)	C9—C10—C11—C12	0.5 (7)
C2—C3—C4—C5	1.1 (6)	C10—C11—C12—C8	0.7 (7)

### N-(Pyridine-2-carbonyl)pyridine-2-carboxamide (1) Hydrogen-bond geometry (Å, º)

**Table:**

*D*—H···*A*	*D*—H	H···*A*	*D*···*A*	*D*—H···*A*
N2—H2···N1	0.90 (5)	2.15 (6)	2.614 (5)	111 (4)
N2—H2···N3	0.90 (5)	2.15 (5)	2.637 (4)	113 (5)
C3—H3···O2^i^	0.95	2.48	3.343 (5)	152
C5—H5···N1^ii^	0.95	2.51	3.393 (5)	154

Symmetry codes: (i) −*x*+1/2, *y*+1/2, *z*−3/2; (ii) −*x*, −*y*+1, *z*−1/2.

### N-(3-Fluoropyridine-2-carbonyl)pyridine-2-carboxamide (2) Crystal data

**Table:**

C_12_H_8_FN_3_O_2_	*D*_x_ = 1.546 Mg m^−^^3^
*M**_r_* = 245.21	Cu *K*α radiation, λ = 1.54184 Å
Orthorhombic, *P**n**a*2_1_	Cell parameters from 2360 reflections
*a* = 16.6058 (10) Å	θ = 3.4–74.8°
*b* = 12.9096 (7) Å	µ = 1.03 mm^−^^1^
*c* = 4.9153 (3) Å	*T* = 100 K
*V* = 1053.71 (11) Å^3^	Block, clear colourless
*Z* = 4	0.26 × 0.10 × 0.05 mm
*F*(000) = 504	

### N-(3-Fluoropyridine-2-carbonyl)pyridine-2-carboxamide (2) Data collection

**Table:**

SuperNova, Dual, Cu at zero, Atlas diffractometer	1798 independent reflections
Radiation source: micro-focus sealed X-ray tube, SuperNova (Cu) X-ray Source	1567 reflections with *I* > 2σ(*I*)
Mirror monochromator	*R*_int_ = 0.054
Detector resolution: 10.4839 pixels mm^-1^	θ_max_ = 75.9°, θ_min_ = 5.3°
ω scans	*h* = −19→20
Absorption correction: gaussian (CrysAlisPro; Rigaku OD, 2015)	*k* = −15→16
*T*_min_ = 0.983, *T*_max_ = 0.995	*l* = −6→5
5774 measured reflections	

### N-(3-Fluoropyridine-2-carbonyl)pyridine-2-carboxamide (2) Refinement

**Table:**

Refinement on *F*^2^	Hydrogen site location: mixed
Least-squares matrix: full	H atoms treated by a mixture of independent and constrained refinement
*R*[*F*^2^ > 2σ(*F*^2^)] = 0.055	*w* = 1/[σ^2^(*F*_o_^2^) + (0.0898*P*)^2^ + 0.4172*P*] where *P* = (*F*_o_^2^ + 2*F*_c_^2^)/3
*wR*(*F*^2^) = 0.152	(Δ/σ)_max_ < 0.001
*S* = 1.03	Δρ_max_ = 0.28 e Å^−^^3^
1798 reflections	Δρ_min_ = −0.28 e Å^−^^3^
176 parameters	Absolute structure: Flack *x* determined using 450 quotients [(*I*^+^)-(*I*^-^)]/[(*I*^+^)+(*I*^-^)] (Parsons *et al.*, 2013)
1 restraint	Absolute structure parameter: 0.2 (3)
Primary atom site location: structure-invariant direct methods	

### N-(3-Fluoropyridine-2-carbonyl)pyridine-2-carboxamide (2) Special details

**Table:**

Geometry. All esds (except the esd in the dihedral angle between two l.s. planes) are estimated using the full covariance matrix. The cell esds are taken into account individually in the estimation of esds in distances, angles and torsion angles; correlations between esds in cell parameters are only used when they are defined by crystal symmetry. An approximate (isotropic) treatment of cell esds is used for estimating esds involving l.s. planes.

### N-(3-Fluoropyridine-2-carbonyl)pyridine-2-carboxamide (2) Fractional atomic coordinates and isotropic or equivalent isotropic displacement parameters (Å^2^)

**Table:**

	*x*	*y*	*z*	*U*_iso_*/*U*_eq_	Occ. (<1)
O2	0.27904 (19)	0.7427 (2)	0.1952 (8)	0.0338 (8)	
O1	0.21184 (17)	0.8809 (3)	0.5995 (8)	0.0359 (8)	
N3	0.4847 (2)	0.7588 (3)	0.3876 (9)	0.0309 (9)	
N2	0.3438 (2)	0.8296 (3)	0.5407 (9)	0.0271 (8)	
N1	0.3932 (2)	0.9540 (3)	0.9257 (8)	0.0269 (8)	
C12	0.4327 (3)	0.6517 (4)	0.0372 (11)	0.0407 (12)	
H12	0.387317	0.626086	−0.059309	0.049*	0.563 (8)
C8	0.4221 (3)	0.7223 (3)	0.2426 (10)	0.0267 (9)	
C9	0.5586 (3)	0.7247 (3)	0.3242 (12)	0.0358 (11)	
H9	0.603043	0.749297	0.427390	0.043*	
C10	0.5730 (3)	0.6555 (4)	0.1156 (11)	0.0393 (11)	
H10	0.626396	0.634525	0.073279	0.047*	
C11	0.5087 (3)	0.6176 (4)	−0.0299 (12)	0.0433 (13)	
H11	0.516473	0.569153	−0.172922	0.052*	
C7	0.3403 (2)	0.7643 (3)	0.3213 (10)	0.0270 (9)	
C6	0.2827 (2)	0.8864 (3)	0.6609 (9)	0.0257 (9)	
C1	0.3139 (2)	0.9587 (3)	0.8767 (10)	0.0247 (9)	
C2	0.2642 (3)	1.0262 (3)	1.0150 (10)	0.0300 (10)	
H2A	0.208315	1.028822	0.973723	0.036*	0.437 (8)
C3	0.2959 (3)	1.0902 (3)	1.2140 (12)	0.0351 (11)	
H3	0.262391	1.137086	1.310847	0.042*	
C4	0.3766 (3)	1.0842 (3)	1.2677 (11)	0.0341 (10)	
H4	0.400053	1.126025	1.405499	0.041*	
C5	0.4234 (3)	1.0164 (3)	1.1188 (10)	0.0302 (9)	
H5	0.479609	1.013741	1.154679	0.036*	
F1B	0.3801 (4)	0.6170 (5)	−0.1240 (16)	0.046 (2)	0.437 (8)
F1A	0.1855 (3)	1.0352 (3)	0.9733 (12)	0.0368 (16)	0.563 (8)
H2	0.395 (3)	0.837 (4)	0.611 (14)	0.044*	

### N-(3-Fluoropyridine-2-carbonyl)pyridine-2-carboxamide (2) Atomic displacement parameters (Å^2^)

**Table:**

	*U*^11^	*U*^22^	*U*^33^	*U*^12^	*U*^13^	*U*^23^
O2	0.0306 (15)	0.0302 (14)	0.0407 (19)	−0.0041 (12)	−0.0077 (16)	−0.0028 (15)
O1	0.0223 (15)	0.0426 (17)	0.043 (2)	−0.0018 (12)	−0.0037 (15)	0.0012 (16)
N3	0.0279 (18)	0.0288 (17)	0.036 (2)	−0.0004 (13)	−0.0008 (17)	−0.0003 (17)
N2	0.0239 (17)	0.0283 (17)	0.029 (2)	−0.0004 (13)	−0.0022 (15)	−0.0039 (15)
N1	0.0249 (17)	0.0281 (16)	0.028 (2)	−0.0030 (13)	−0.0019 (15)	−0.0021 (15)
C12	0.055 (3)	0.034 (2)	0.033 (3)	0.006 (2)	−0.009 (3)	−0.003 (2)
C8	0.029 (2)	0.0221 (16)	0.029 (2)	−0.0005 (14)	−0.0027 (18)	0.0019 (17)
C9	0.033 (2)	0.028 (2)	0.047 (3)	0.0026 (17)	0.003 (2)	−0.001 (2)
C10	0.045 (2)	0.030 (2)	0.043 (3)	0.0102 (19)	0.012 (2)	0.005 (2)
C11	0.061 (3)	0.036 (2)	0.033 (3)	0.010 (2)	0.004 (3)	−0.004 (2)
C7	0.031 (2)	0.0227 (18)	0.027 (2)	−0.0036 (15)	−0.003 (2)	0.0033 (17)
C6	0.0198 (18)	0.0282 (18)	0.029 (3)	−0.0029 (15)	−0.0016 (17)	0.0038 (19)
C1	0.0218 (18)	0.0222 (16)	0.030 (2)	−0.0014 (14)	−0.0005 (19)	0.0011 (16)
C2	0.028 (2)	0.0232 (18)	0.039 (3)	0.0010 (15)	0.006 (2)	0.0045 (19)
C3	0.043 (3)	0.0250 (19)	0.037 (3)	−0.0003 (17)	0.009 (2)	−0.003 (2)
C4	0.044 (3)	0.0276 (19)	0.030 (2)	−0.0053 (18)	0.005 (2)	−0.002 (2)
C5	0.030 (2)	0.0301 (19)	0.031 (2)	−0.0040 (16)	−0.001 (2)	−0.002 (2)
F1B	0.036 (4)	0.053 (4)	0.050 (5)	−0.002 (3)	−0.012 (3)	−0.025 (4)
F1A	0.020 (2)	0.028 (2)	0.062 (4)	0.0007 (16)	0.002 (2)	0.001 (2)

### N-(3-Fluoropyridine-2-carbonyl)pyridine-2-carboxamide (2) Geometric parameters (Å, º)

**Table:**

O2—C7	1.224 (5)	C9—C10	1.381 (7)
O1—C6	1.217 (5)	C10—H10	0.9500
N3—C8	1.346 (6)	C10—C11	1.376 (8)
N3—C9	1.340 (6)	C11—H11	0.9500
N2—C7	1.370 (6)	C6—C1	1.505 (6)
N2—C6	1.384 (6)	C1—C2	1.380 (6)
N2—H2	0.92 (6)	C2—H2A	0.9500
N1—C1	1.341 (5)	C2—C3	1.384 (7)
N1—C5	1.342 (6)	C2—F1A	1.327 (6)
C12—H12	0.9500	C3—H3	0.9500
C12—C8	1.372 (7)	C3—C4	1.369 (7)
C12—C11	1.376 (7)	C4—H4	0.9500
C12—F1B	1.262 (8)	C4—C5	1.381 (6)
C8—C7	1.511 (6)	C5—H5	0.9500
C9—H9	0.9500		
			
C9—N3—C8	118.0 (4)	O2—C7—C8	122.4 (4)
C7—N2—C6	129.1 (4)	N2—C7—C8	112.6 (3)
C7—N2—H2	113 (4)	O1—C6—N2	124.9 (4)
C6—N2—H2	118 (4)	O1—C6—C1	122.9 (4)
C1—N1—C5	117.9 (4)	N2—C6—C1	112.2 (3)
C8—C12—H12	119.8	N1—C1—C6	115.9 (3)
C8—C12—C11	120.5 (5)	N1—C1—C2	121.9 (4)
C11—C12—H12	119.8	C2—C1—C6	122.2 (4)
F1B—C12—C8	127.5 (6)	C1—C2—H2A	120.1
F1B—C12—C11	111.7 (6)	C1—C2—C3	119.8 (4)
N3—C8—C12	121.5 (4)	C3—C2—H2A	120.1
N3—C8—C7	115.7 (4)	F1A—C2—C1	124.6 (5)
C12—C8—C7	122.8 (4)	F1A—C2—C3	115.6 (4)
N3—C9—H9	118.5	C2—C3—H3	120.8
N3—C9—C10	122.9 (5)	C4—C3—C2	118.4 (4)
C10—C9—H9	118.5	C4—C3—H3	120.8
C9—C10—H10	120.6	C3—C4—H4	120.5
C11—C10—C9	118.8 (5)	C3—C4—C5	119.0 (4)
C11—C10—H10	120.6	C5—C4—H4	120.5
C12—C11—H11	120.9	N1—C5—C4	123.0 (4)
C10—C11—C12	118.3 (5)	N1—C5—H5	118.5
C10—C11—H11	120.9	C4—C5—H5	118.5
O2—C7—N2	125.0 (4)		
			
O1—C6—C1—N1	178.5 (4)	C11—C12—C8—C7	178.6 (4)
O1—C6—C1—C2	−1.9 (7)	C7—N2—C6—O1	6.0 (7)
N3—C8—C7—O2	175.2 (4)	C7—N2—C6—C1	−172.9 (4)
N3—C8—C7—N2	−3.2 (5)	C6—N2—C7—O2	−2.1 (7)
N3—C9—C10—C11	−1.7 (8)	C6—N2—C7—C8	176.3 (4)
N2—C6—C1—N1	−2.6 (5)	C6—C1—C2—C3	179.2 (4)
N2—C6—C1—C2	177.0 (4)	C6—C1—C2—F1A	−0.4 (7)
N1—C1—C2—C3	−1.2 (7)	C1—N1—C5—C4	0.2 (6)
N1—C1—C2—F1A	179.2 (4)	C1—C2—C3—C4	0.0 (7)
C12—C8—C7—O2	−4.7 (6)	C2—C3—C4—C5	1.2 (7)
C12—C8—C7—N2	176.8 (4)	C3—C4—C5—N1	−1.3 (7)
C8—N3—C9—C10	1.0 (7)	C5—N1—C1—C6	−179.3 (4)
C8—C12—C11—C10	0.6 (8)	C5—N1—C1—C2	1.1 (6)
C9—N3—C8—C12	0.6 (7)	F1B—C12—C8—N3	−175.0 (6)
C9—N3—C8—C7	−179.4 (4)	F1B—C12—C8—C7	5.0 (9)
C9—C10—C11—C12	0.9 (7)	F1B—C12—C11—C10	175.1 (6)
C11—C12—C8—N3	−1.3 (7)	F1A—C2—C3—C4	179.6 (5)

### N-(3-Fluoropyridine-2-carbonyl)pyridine-2-carboxamide (2) Hydrogen-bond geometry (Å, º)

**Table:**

*D*—H···*A*	*D*—H	H···*A*	*D*···*A*	*D*—H···*A*
N2—H2···N1	0.92 (5)	2.16 (6)	2.614 (6)	109 (4)
N2—H2···N3	0.92 (5)	2.11 (6)	2.622 (5)	114 (5)
C3—H3···O2^i^	0.95	2.43	3.320 (6)	156
C3—H3···F1*B*^i^	0.95	2.40	3.049 (8)	125
C5—H5···N1^ii^	0.95	2.53	3.420 (6)	156
C10—H10···F1*A*^iii^	0.95	2.45	3.169 (7)	132

Symmetry codes: (i) −*x*+1/2, *y*+1/2, *z*+3/2; (ii) −*x*+1, −*y*+2, *z*+1/2; (iii) *x*+1/2, −*y*+3/2, *z*−1.

### 3-Fluoro-N-(3-fluoropyridine-2-carbonyl)pyridine-2-carboxamide (3) Crystal data

**Table:**

C_12_H_7_F_2_N_3_O_2_	*F*(000) = 536
*M**_r_* = 263.21	*D*_x_ = 1.660 Mg m^−^^3^
Monoclinic, *I*2/*a*	Cu *K*α radiation, λ = 1.54184 Å
*a* = 6.7062 (3) Å	Cell parameters from 1899 reflections
*b* = 14.1190 (5) Å	θ = 5.0–74.9°
*c* = 11.2074 (5) Å	µ = 1.22 mm^−^^1^
β = 97.140 (4)°	*T* = 100 K
*V* = 1052.94 (8) Å^3^	Block, clear colourless
*Z* = 4	0.11 × 0.09 × 0.06 mm

### 3-Fluoro-N-(3-fluoropyridine-2-carbonyl)pyridine-2-carboxamide (3) Data collection

**Table:**

SuperNova, Dual, Cu at zero, Atlas diffractometer	1083 independent reflections
Radiation source: micro-focus sealed X-ray tube, SuperNova (Cu) X-ray Source	856 reflections with *I* > 2σ(*I*)
Mirror monochromator	*R*_int_ = 0.069
Detector resolution: 10.4839 pixels mm^-1^	θ_max_ = 75.4°, θ_min_ = 5.1°
ω scans	*h* = −7→8
Absorption correction: gaussian (CrysAlisPro; Rigaku OD, 2015)	*k* = −17→17
*T*_min_ = 0.993, *T*_max_ = 0.996	*l* = −14→13
5200 measured reflections	

### 3-Fluoro-N-(3-fluoropyridine-2-carbonyl)pyridine-2-carboxamide (3) Refinement

**Table:**

Refinement on *F*^2^	Primary atom site location: structure-invariant direct methods
Least-squares matrix: full	Hydrogen site location: mixed
*R*[*F*^2^ > 2σ(*F*^2^)] = 0.055	H atoms treated by a mixture of independent and constrained refinement
*wR*(*F*^2^) = 0.161	*w* = 1/[σ^2^(*F*_o_^2^) + (0.0954*P*)^2^ + 0.7955*P*] where *P* = (*F*_o_^2^ + 2*F*_c_^2^)/3
*S* = 1.04	(Δ/σ)_max_ < 0.001
1083 reflections	Δρ_max_ = 0.29 e Å^−^^3^
88 parameters	Δρ_min_ = −0.32 e Å^−^^3^
0 restraints	

### 3-Fluoro-N-(3-fluoropyridine-2-carbonyl)pyridine-2-carboxamide (3) Special details

**Table:**

Geometry. All esds (except the esd in the dihedral angle between two l.s. planes) are estimated using the full covariance matrix. The cell esds are taken into account individually in the estimation of esds in distances, angles and torsion angles; correlations between esds in cell parameters are only used when they are defined by crystal symmetry. An approximate (isotropic) treatment of cell esds is used for estimating esds involving l.s. planes.

### 3-Fluoro-N-(3-fluoropyridine-2-carbonyl)pyridine-2-carboxamide (3) Fractional atomic coordinates and isotropic or equivalent isotropic displacement parameters (Å^2^)

**Table:**

	*x*	*y*	*z*	*U*_iso_*/*U*_eq_	
F1	0.1012 (3)	0.49942 (9)	0.14190 (14)	0.0349 (5)	
O1	0.1649 (3)	0.53996 (11)	0.37977 (16)	0.0296 (5)	
N1	0.0596 (3)	0.29674 (13)	0.33292 (17)	0.0204 (5)	
N2	0.250000	0.41071 (19)	0.500000	0.0218 (6)	
C2	0.0612 (4)	0.41086 (16)	0.1771 (2)	0.0245 (6)	
C1	0.0945 (3)	0.38567 (15)	0.2979 (2)	0.0217 (5)	
C5	−0.0104 (3)	0.23375 (15)	0.2494 (2)	0.0208 (5)	
H5	−0.037842	0.171355	0.274533	0.025*	
C4	−0.0453 (3)	0.25475 (16)	0.1273 (2)	0.0233 (5)	
H4	−0.094044	0.207588	0.070604	0.028*	
C3	−0.0075 (4)	0.34557 (16)	0.0902 (2)	0.0247 (6)	
H3	−0.028177	0.362470	0.007558	0.030*	
C6	0.1711 (3)	0.45473 (15)	0.3944 (2)	0.0213 (5)	
H2	0.250000	0.351 (3)	0.500000	0.026*	

### 3-Fluoro-N-(3-fluoropyridine-2-carbonyl)pyridine-2-carboxamide (3) Atomic displacement parameters (Å^2^)

**Table:**

	*U*^11^	*U*^22^	*U*^33^	*U*^12^	*U*^13^	*U*^23^
F1	0.0551 (11)	0.0191 (7)	0.0292 (9)	−0.0062 (6)	−0.0002 (7)	0.0071 (6)
O1	0.0404 (10)	0.0157 (8)	0.0316 (10)	0.0016 (7)	0.0006 (8)	0.0023 (7)
N1	0.0190 (9)	0.0171 (9)	0.0254 (10)	0.0017 (7)	0.0038 (7)	0.0014 (7)
N2	0.0254 (13)	0.0141 (12)	0.0262 (15)	0.000	0.0042 (11)	0.000
C2	0.0262 (11)	0.0165 (10)	0.0309 (13)	0.0012 (9)	0.0042 (10)	0.0048 (9)
C1	0.0203 (11)	0.0167 (11)	0.0278 (12)	0.0022 (8)	0.0023 (9)	0.0021 (9)
C5	0.0188 (10)	0.0174 (10)	0.0270 (12)	0.0007 (8)	0.0058 (9)	0.0008 (8)
C4	0.0213 (11)	0.0223 (11)	0.0258 (12)	0.0025 (8)	0.0014 (9)	−0.0022 (9)
C3	0.0276 (11)	0.0239 (12)	0.0222 (12)	0.0039 (9)	0.0014 (10)	0.0028 (9)
C6	0.0222 (11)	0.0162 (10)	0.0259 (12)	0.0014 (8)	0.0051 (9)	0.0009 (9)

### 3-Fluoro-N-(3-fluoropyridine-2-carbonyl)pyridine-2-carboxamide (3) Geometric parameters (Å, º)

**Table:**

F1—C2	1.348 (2)	C2—C3	1.378 (3)
O1—C6	1.214 (3)	C1—C6	1.498 (3)
N1—C1	1.344 (3)	C5—H5	0.9500
N1—C5	1.334 (3)	C5—C4	1.391 (3)
N2—C6^i^	1.383 (3)	C4—H4	0.9500
N2—C6	1.383 (3)	C4—C3	1.381 (3)
N2—H2	0.85 (4)	C3—H3	0.9500
C2—C1	1.391 (3)		
			
C5—N1—C1	118.5 (2)	N1—C5—C4	123.4 (2)
C6^i^—N2—C6	126.6 (3)	C4—C5—H5	118.3
C6^i^—N2—H2	116.71 (13)	C5—C4—H4	120.7
C6—N2—H2	116.71 (13)	C3—C4—C5	118.6 (2)
F1—C2—C1	120.5 (2)	C3—C4—H4	120.7
F1—C2—C3	118.4 (2)	C2—C3—C4	117.8 (2)
C3—C2—C1	121.1 (2)	C2—C3—H3	121.1
N1—C1—C2	120.7 (2)	C4—C3—H3	121.1
N1—C1—C6	117.0 (2)	O1—C6—N2	124.3 (2)
C2—C1—C6	122.3 (2)	O1—C6—C1	123.0 (2)
N1—C5—H5	118.3	N2—C6—C1	112.68 (19)
			
F1—C2—C1—N1	−178.3 (2)	C1—C2—C3—C4	1.0 (4)
F1—C2—C1—C6	1.3 (4)	C5—N1—C1—C2	−1.0 (3)
F1—C2—C3—C4	179.1 (2)	C5—N1—C1—C6	179.41 (19)
N1—C1—C6—O1	−162.6 (2)	C5—C4—C3—C2	−0.6 (3)
N1—C1—C6—N2	17.9 (3)	C3—C2—C1—N1	−0.2 (4)
N1—C5—C4—C3	−0.6 (3)	C3—C2—C1—C6	179.3 (2)
C2—C1—C6—O1	17.8 (4)	C6^i^—N2—C6—O1	1.68 (18)
C2—C1—C6—N2	−161.7 (2)	C6^i^—N2—C6—C1	−178.8 (2)
C1—N1—C5—C4	1.4 (3)		

Symmetry code: (i) −*x*+1/2, *y*, −*z*+1.

### 3-Fluoro-N-(3-fluoropyridine-2-carbonyl)pyridine-2-carboxamide (3) Hydrogen-bond geometry (Å, º)

**Table:**

*D*—H···*A*	*D*—H	H···*A*	*D*···*A*	*D*—H···*A*
N2—H2···N1	0.84 (4)	2.27 (2)	2.671 (2)	110 (1)
N2—H2···N1^i^	0.84 (4)	2.27 (2)	2.671 (2)	110 (1)
C4—H4···O1^ii^	0.95	2.49	3.135 (3)	125
C5—H5···O1^ii^	0.95	2.61	3.207 (3)	122
C3—H3···F1^iii^	0.95	2.58	3.398 (3)	145
C5—H5···F1^ii^	0.95	2.66	3.604 (3)	176

Symmetry codes: (i) −*x*+1/2, *y*, −*z*+1; (ii) −*x*, *y*−1/2, −*z*+1/2; (iii) −*x*, −*y*+1, −*z*.
